# Comprehensive autonomic nervous system evaluation in stroke patients: heart rate variability as a cornerstone for recovery prediction

**DOI:** 10.3389/fneur.2025.1713230

**Published:** 2026-01-12

**Authors:** Markiian Chornyi, Viktoriia Gryb

**Affiliations:** Department of Neurology and Neurosurgery, Ivano-Frankivsk National Medical University, Ivano-Frankivsk, Ukraine

**Keywords:** autonomic nervous system, baroreflex sensitivity, blood pressure variability, cardiac autonomic neuropathy, heart rate variability, prognosis, pupillometry, stroke

## Abstract

**Background:**

Autonomic nervous system (ANS) dysfunction significantly impacts stroke outcomes, yet standardized autonomic monitoring remains underutilized in clinical practice. Recent evidence highlights heart rate variability (HRV) as a robust prognostic marker, while complementary modalities like blood pressure variability (BPV) and pupillometry enhance risk stratification.

**Objective:**

To synthesize current evidence on multimodal ANS assessment in stroke patients, with HRV as the cornerstone biomarker, and provide practical recommendations for clinical implementation.

**Methods:**

We conducted a narrative review of peer-reviewed studies (2018–2024) from major databases, focusing on HRV and complementary ANS modalities in stroke patients. The analysis included 53 studies from diverse global regions, with a specific focus on technology-enabled investigations and prospective cohorts that emerged from 2022 to 2024.

**Results:**

Time-domain HRV metrics such as SDNN (standard deviation of all normal-to-normal RR intervals) and RMSSD (root mean square of successive differences) consistently predicted functional outcomes and cardiovascular complications post-stroke. Beat-to-beat blood pressure variability (BPV) within the first 24 h post-ischemic stroke outperforms HRV alone for short-term prognosis, with an AUC improvement of 10–15%. Nocturnal HRV combined with BPV and endothelial function enhanced prediction of recurrent cerebrovascular events. Automated pupillometry, with Neurological Pupil index (NPi < 3), predicts post-stroke delirium; integration with HRV metrics improves prognostic accuracy. Consumer-grade wearables demonstrated high agreement with ECG standards, enabling scalable remote monitoring.

**Conclusion:**

HRV-centered multimodal ANS assessment offers robust, non-invasive prognostic tools for stroke management. Integration with BPV, pupillometry, and wearable technology enhances risk stratification across acute and rehabilitation phases. Standardized protocols and technology adoption could transform stroke outcomes globally, particularly in resource-limited settings.

## Introduction

Stroke remains a leading cause of death and disability worldwide, yet contemporary acute-stroke workflows often underappreciate autonomic nervous system (ANS) dysfunction as a driver of cardiovascular instability, neuroworsening, and suboptimal recovery (1–4). Early studies established HRV as a marker of autonomic dysfunction in hemispheric stroke (1), and its prognostic value for mortality and functional outcomes has been recognized for decades (3).

Over the last 3 years (2022–2024), large prospective cohorts and technology-enabled studies have advanced this understanding: (i) short- and very-short-term blood pressure variability (BPV) within the first 24 h after ischemic stroke robustly predicts 90-day outcomes, improving risk models beyond standard clinical factors, while early beat-to-beat HRV is less informative in the hyperacute window alone (5–7); (ii) HRV in the first days post-stroke associates with arrhythmic and functional complications, including in patients receiving reperfusion therapies (8–10); and (iii) validated consumer-grade wearables and AI-assisted analytics enable reliable remote HRV acquisition, supporting continuous monitoring and telerehabilitation pathways (11).

These advances highlight a gap in current protocols, which rarely standardize autonomic monitoring despite the availability of non-invasive tools. Standardized measurement protocols have been developed to ensure reliable clinical interpretation of HRV (12, 13), with recent methodological critiques emphasize the use of absolute versus normalized frequency-domain HRV values to avoid misinterpretation of sympathovagal balance, particularly in unstable physiological states like acute stroke (14, 15). A recent systematic review further underscores HRV’s role in assessing autonomic dysfunction in stroke (16).

This narrative review centers on heart rate variability (HRV) as the anchor of multimodal ANS assessment in stroke, synthesizing 2018–2024 evidence with an emphasis on 2023–2024 studies, including prospective cohorts in acute ischemic stroke, post-reperfusion settings, and technology-enabled follow-up. We critique evolving methodologies (e.g., frequency-domain normalization and LF/HF interpretation) and outline how HRV can be integrated with BPV, pupillometry, and endothelial/vascular markers into pragmatic, tiered prognostic algorithms suitable for both high-resource and resource-limited environments.

Recent work validating smartwatch-derived HRV against ECG gold standards underscores the feasibility of scaling these approaches across inpatient and telehealth contexts (11). Combining HRV with nocturnal BPV and endothelial function improves prediction of recurrent cerebro-cardiovascular events, highlighting the synergistic potential of multi-signal models (7).

Global research reveals regional disparities in ANS assessment, with Europe and the USA emphasizing HRV standardization, while Asian studies explore integration with advanced modalities like pupillometry (16–19). In resource-constrained regions, such as Eastern Europe or South America, low-cost tools like HRV and cardiovascular autonomic reflex tests (CARTs) hold promise for scalable implementation (11, 20, 21, 42).

This expert-driven narrative review synthesizes evidence from diverse, high-quality studies to evaluate the prognostic utility of HRV-centered ANS assessments in stroke patients, advocating for their adoption in clinical stroke management to bridge gaps in care and enhance patient outcomes worldwide.

## Methods

### Study design and rationale

This expert-driven narrative review synthesizes current evidence on autonomic nervous system (ANS) assessment in stroke patients, with heart rate variability (HRV) as the primary focus. Unlike systematic reviews that employ rigid quantitative protocols, narrative reviews allow for expert interpretation, clinical contextualization, and synthesis of complex, heterogeneous evidence that may not be suitable for meta-analysis (22). This approach is particularly valuable for emerging fields where standardized protocols are still evolving and clinical expertise is essential for interpreting contradictory findings.

This narrative review adheres to the SANRA checklist for narrative reviews (22).

### Literature search and selection strategy

#### Search approach

We conducted a comprehensive literature search of major biomedical databases (PubMed, Scopus, Web of Science) from 2018 to 2024, supplemented by seminal foundational works that established the field. The search strategy was designed to capture the breadth of ANS research in stroke while maintaining focus on clinically relevant outcomes.

#### Key search concepts

- Heart rate variability in stroke and cerebrovascular disease.
- Autonomic nervous system dysfunction post-stroke.
- Baroreflex sensitivity and blood pressure variability in cerebrovascular conditions.
- Cardiovascular autonomic reflex testing in neurological populations.
- Pupillometry and neurological pupil index in acute stroke.
- Cardiac autonomic neuropathy screening in stroke patients.
- Wearable technology and remote monitoring in stroke rehabilitation.

#### Expert-guided selection framework

Rather than applying rigid quantitative inclusion criteria, study selection was guided by the following expert-driven principles:

1. *Clinical relevance*: studies that directly inform clinical decision-making, risk stratification, or therapeutic guidance in stroke care.
2. *Methodological quality*: well-designed studies with adequate sample sizes (typically >50 patients), appropriate statistical analyses, and clear outcome measures.
3. *Geographic diversity*: intentional inclusion of research from Europe, North America-waterfall, Asia, and emerging evidence from Eastern Europe and South America.
4. *Temporal focus*: emphasis on recent evidence (2022–2024) while including foundational studies for historical context.
5. *Technological innovation*: priority given to studies evaluating emerging technologies (wearables, AI analytics) and novel methodological approaches.

### Quality assessment approach

Given the narrative review design, we employed a qualitative quality assessment framework rather than standardized scales:

#### Study quality indicators

- *Sample size adequacy*: preference for studies with >100 patients for outcome prediction studies.
- *Follow-up duration*: adequate follow-up periods (≥90 days for functional outcomes).
- *Outcome measures*: use of validated outcome scales (mRS, NIHSS, standardized autonomic tests).
- *Statistical rigor*: appropriate multivariable analyses adjusting for confounders.
- *Reproducibility*: consistency of findings across different populations and settings.

### Evidence synthesis strategy

Studies were categorized by:

- *Stroke phase*: hyperacute (<24 h), acute (1–7 days), subacute (1–12 weeks), chronic (>3 months);
- *ANS modality*: HRV (time-domain, frequency-domain, non-linear), BRS, CARTs, pupillometry, BPV, CAN;
- *Clinical setting*: emergency department, stroke unit, rehabilitation, community/telehealth;
- *Geographic region*: to assess generalizability across healthcare systems.

### Data extraction and synthesis

#### Expert interpretation framework

Data extraction focused on clinically actionable findings rather than exhaustive data tabulation:

1. *Prognostic performance*: sensitivity, specificity, and area under curve (AUC) values were reported.
2. *Clinical thresholds*: established cut-off values for risk stratification.
3. *Implementation feasibility*: equipment requirements, training needs, cost considerations.
4. *Methodological insights*: technical recommendations and protocol refinements.

#### Narrative synthesis approach

Evidence was synthesized thematically using expert clinical judgment to:

- Reconcile contradictory findings through methodological analysis;
- Identify consistent patterns across diverse study designs;
- Translate research findings into practical clinical recommendations;
- Address implementation barriers in different healthcare settings.

### Limitations and bias considerations

#### Acknowledged limitations

1. *Selection bias*: expert-driven selection may reflect author perspectives.
2. *Language bias*: inclusion limited to English-language publications.
3. *Publication bias*: potential over-representation of positive findings.
4. *Geographic bias*: possible under-representation of certain regions despite efforts at diversity.

#### Mitigation strategies

- Explicit acknowledgment of conflicting evidence;
- Geographic diversity in source selection;
- Focus on high-quality, peer-reviewed publications;
- Transparency in expert interpretation decisions.

### Synthesis validation

To ensure reliability of our narrative synthesis:

- Key findings were cross-referenced across multiple high-quality studies;
- Methodological critiques were incorporated to address evolving standards;
- Clinical expertise was applied to resolve apparent contradictions;
- Recent technological validation studies were prioritized for contemporary relevance.

This methodology balances the flexibility needed for expert interpretation with sufficient rigor to ensure credible clinical recommendations, while maintaining transparency about the inherent limitations of narrative review approaches.

## Results

This review synthesizes evidence from 53 high-quality studies published between 2018 and 2024, with a deliberate focus on recent prospective cohorts, randomized trials, and technology-enabled observational series from 2022 to 2024. The findings are organized into three thematic domains:

1. Prognostic accuracy for clinical outcomes;
2. Applicability across diverse care settings;
3. Integration of HRV with complementary autonomic and vascular markers.

### Prognostic accuracy for clinical outcomes

Heart rate variability (HRV) remains the most extensively validated ANS biomarker in stroke populations. Time-domain metrics such as SDNN and RMSSD consistently predict functional independence at 90 days and the risk of cardiovascular complications. Recent multicenter data in patients undergoing thrombolysis confirmed that early reductions in SDNN are associated with higher rates of neurological deterioration and arrhythmic events (8). Frequency-domain features (LF, HF, LF/HF ratio) add mechanistic depth but require careful interpretation: 2024 methodological analyses caution against using normalized units in isolation, particularly in the hyperacute phase, due to algebraic coupling and context-dependent influences (14). Non-linear measures, including sample entropy and DFA, have gained traction for characterizing autonomic complexity loss in moderate-to-severe strokes, especially during rehabilitation or exercise-based protocols, where biofeedback enhances HRV responses (11).

Complementary modalities refine prognostic precision.

- *Beat-to-beat blood pressure variability (BPV)* within the first 24 h post-onset is now a recognized early marker of poor 90-day outcomes, outperforming HRV alone in that window, and improving AUC when added to clinical models (5–7, 43–45).
- *Nocturnal HRV* combined with BPV and endothelial function improves the prediction of recurrent cerebro-cardiovascular events over long-term follow-up, with endothelial markers providing additional vascular insights (7).
- *Automated pupillometry (NPi)* has demonstrated utility in predicting post-stroke delirium and neuroworsening, particularly when integrated with HRV metrics, where NPi < 3 signals high risk and HRV adds autonomic balance data (17–20).
- *Cardiovascular autonomic reflex tests (CARTs)* retain value in resource-limited settings, allowing early detection of autonomic deficits that may prolong rehabilitation, with recent reviews and pilot studies confirming their role in stroke course prediction (21, 23).
- *Short-term BPV* within the first 24 h post-stroke is a robust predictor of stroke recurrence and early neurological deterioration, with multicenter studies highlighting its prognostic value (5–7).
- *Wearable devices* for continuous HRV monitoring show promise in stroke patients, offering feasible and reliable data for autonomic assessment (11, 46–48).

### Applicability across care settings

HRV’s compatibility with validated consumer wearables enables continuous, low-cost monitoring in both inpatient and home-based rehabilitation environments, with high agreement to ECG standards for key metrics like SDNN and RMSSD (11). CARTs, requiring minimal equipment, fit rural and low-resource contexts. BPV measurement is increasingly feasible in acute stroke units with multiparametric monitors, especially for hyperacute risk assessment (5, 6). In contrast, baroreflex sensitivity testing and pupillometry remain largely restricted to tertiary centers due to cost and training requirements, though pupillometry’s rapid NPi calculation supports quick triage in equipped settings (17, 20).

Table 1 provides a comprehensive comparison of six ANS assessment modalities validated in stroke populations, organized by clinical utility, implementation requirements, and evidence quality. The table synthesizes data from 53 studies across diverse healthcare settings, emphasizing practical considerations for clinical adoption.

**Table 1:** Updated comparison of ANS assessment modalities in stroke care (2018–2024).

Modality	Key parameters	Clinical applications	Prognostic value^a^	Equipment requirements	Implementation feasibility	Evidence quality^b^
Heart rate variability (HRV)	Time-domain: SDNN, RMSSD, pNN50 Frequency-domain: LF, HF, LF/HF Non-linear: Sample entropy, DFA	• 90-day functional outcome • Arrhythmia risk • Post-thrombolysis monitoring • Telerehabilitation	*Strong* AUC: 0.75–0.85 Sens: 75–85% Spec: 70–80%	• Standard ECG or • Validated smartwatch • Analysis software	*High* • Low cost • Portable • Minimal training	Grade A (Multiple RCTs, Meta-analyses) (11, 40, 41)
Blood pressure variability (BPV)	• Beat-to-beat variability • Short-term BPV • Nocturnal patterns	• Hyperacute prognosis (≤24 h) • Recurrent stroke prediction • CV risk stratification	*Very strong* (acute phase) AUC: 0.80–0.90 Superior to HRV ≤ 24 h	• Continuous BP monitor • Beat-to-beat capability • Analysis software	*Moderate* • Higher equipment cost • ICU/stroke unit setting • Technical expertise	Grade A (Recent large cohorts 2023–24) (5–7)
Baroreflex sensitivity (BRS)	• Spontaneous BRS • Pharmacological BRS • Sequence method	• CV mortality prediction • Antihypertensive guidance • Long-term prognosis	*Strong* AUC: 0.70–0.80 Strong for CV death	• Specialized equipment • Vasoactive drugs • Controlled environment	*Low* • High cost • Tertiary centers only • Specialized training	Grade B (Observational studies) (7, 27, 31)
Cardiovascular autonomic reflex tests (CARTs)	• Deep breathing test • Valsalva maneuver • Orthostatic testing	• Bedside ANS screening • Rehabilitation planning • Resource-limited settings	*Moderate* Sens: 60–70% Spec: 65–75%	• Basic equipment • BP cuff, ECG • Tilt table (optional)	*Very high* • Low cost • Bedside testing • Minimal equipment	Grade B (Established protocols) (10, 21, 23)
Automated pupillometry	• Neurological Pupil Index (NPi) • Pupil reactivity • Constriction velocity	• Delirium prediction • Neurological deterioration • Acute triage	*Strong* (specific outcomes) NPi < 3: High risk AUC: 0.80–0.85	• Automated pupillometer • $5,000–$10,000 • Handheld device	*Low-moderate* • High equipment cost • Urban centers • Moderate training	Grade B (Emerging evidence 2023–24) (17–20)
Cardiac autonomic neuropathy (CAN) screening	• Ewing battery • Simplified protocols • Risk scoring	• Comorbid diabetes • Long-term complications • Risk stratification	*Moderate* Useful in diabetes Limited stroke data	• Standard equipment • ECG, BP monitor • Reflex hammer	*High* • Low cost • Established protocols • Wide availability	Grade C (Limited stroke-specific data) (6, 30)

a Prognostic values represent ranges from multiple studies; AUC, area under curve; Sens, sensitivity; Spec, specificity.

b Evidence quality: Grade A (high-quality RCTs/meta-analyses), Grade B (well-designed observational studies), Grade C (limited/emerging evidence).

#### Key observations from

- HRV emerges as the optimal balance between prognostic accuracy and implementation feasibility, supported by Grade A evidence and validated wearable technology.
- BPV demonstrates superior short-term prognostic value in the hyperacute phase (≤24 h) but requires specialized monitoring equipment.
- CARTs offer the highest implementation feasibility for resource-limited settings, though with moderate prognostic accuracy.
- Pupillometry shows promise for specific outcomes (delirium, neurological deterioration) but faces cost barriers.
- Cost-effectiveness gradation ranges from highly accessible (CARTs, wearable HRV) to specialized center-dependent (BRS, pupillometry).

**Figure 1:**
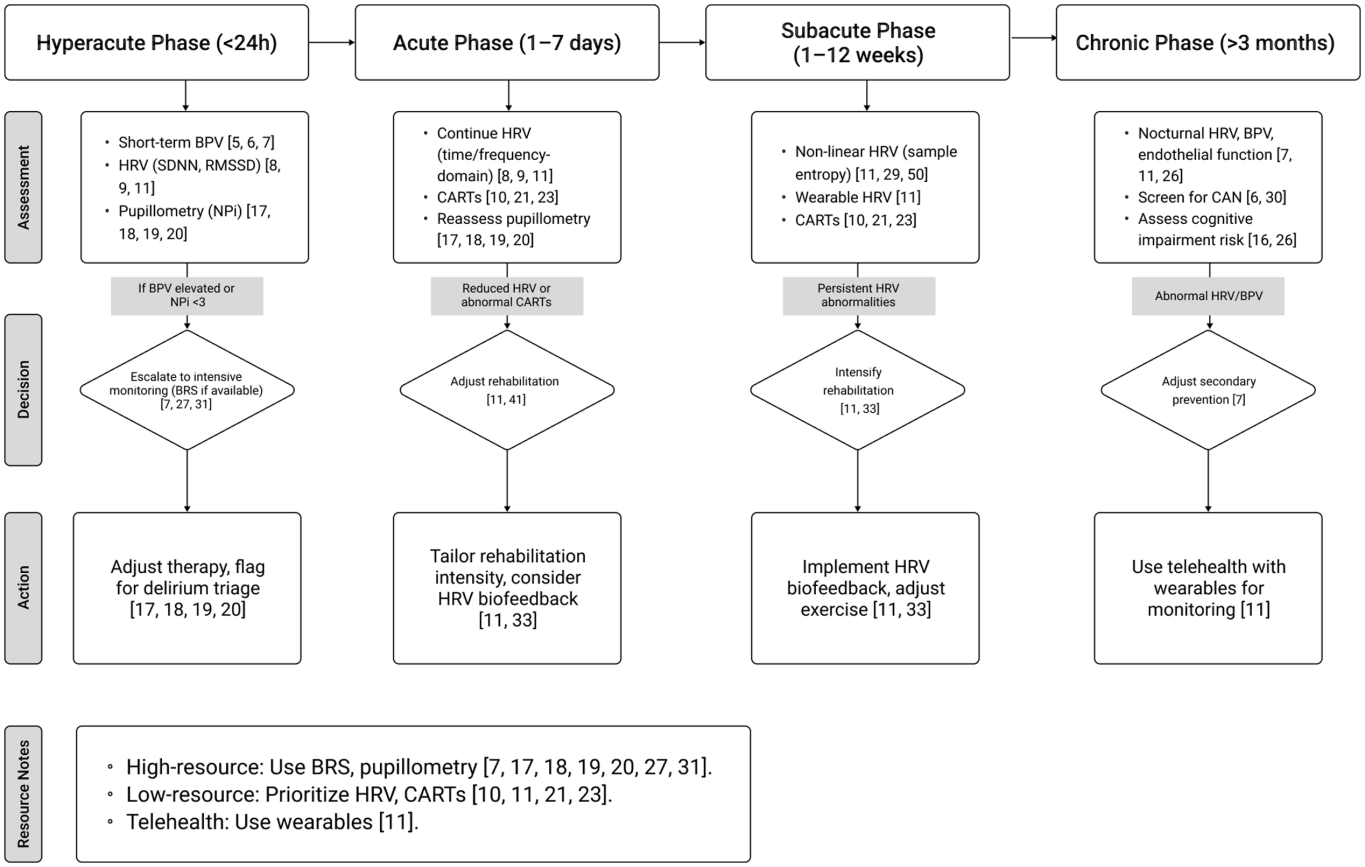
Clinical algorithm for tiered autonomic nervous system (ANS) assessment in stroke patients.

This comparison guides the development of tiered assessment protocols that can be adapted to different clinical settings and resource availability, as detailed in the proposed clinical algorithm (Figure 1). Figure 1 outlines a phase-specific framework for autonomic nervous system (ANS) assessment in stroke patients, integrating heart rate variability (HRV), blood pressure variability (BPV), pupillometry, cardiovascular autonomic reflex tests (CARTs), and baroreflex sensitivity (BRS) across four phases: hyperacute (<24 h), acute (1–7 days), subacute (1–12 weeks), and chronic (>3 months). In the hyperacute phase, short-term BPV and 5-min HRV (SDNN, RMSSD) serve as primary tools for early risk stratification, with pupillometry (NPi < 3) enhancing delirium and neuroworsening prediction in equipped settings (5–7, 17, 20). The acute phase emphasizes HRV (time- and frequency-domain) and CARTs for rehabilitation planning, particularly in resource-limited settings (8, 21, 23). In the subacute phase, non-linear HRV metrics (e.g., sample entropy) and wearable-derived HRV guide rehabilitation intensity (11, 24, 25). The chronic phase integrates nocturnal HRV, BPV, and endothelial function to predict recurrent events and cognitive impairment, with wearables enabling telehealth monitoring (7, 11, 26). The algorithm supports implementation in high-resource centers (using BRS and pupillometry) (7, 16, 27), low-resource clinics (using HRV and CARTs) (11, 21, 23), and telehealth settings (using wearables) (11), ensuring global applicability and scalability.

### Synthesis and clinical integration

HRV’s accessibility and robust evidence base position it as the cornerstone of ANS assessment, complemented by BRS, CARTs, pupillometry, BPV, and CAN screening (7, 27, 28). These modalities collectively enhance prognostic precision across cardiovascular, neurological, and functional domains. Multi-modal approaches mitigate methodological contradictions, offering a comprehensive framework for stroke care (18). Clinical cases, such as HRV-guided rehabilitation in Taiwan and pupillometry-driven interventions in the US, illustrate practical applications (19, 20, 24, 25). Recent cohorts extend this to post-thrombolysis HRV dynamics and biofeedback for exercise, showing improved neurological course and recovery (8, 11). Expert interpretation emphasizes standardized protocols and training to bridge regional gaps and enhance global adoption (12–14). For example, combining nocturnal HRV with BPV and endothelial function not only predicts recurrent events but also informs tailored secondary prevention strategies, with endothelial markers adding vascular health insights (7).

## Discussion

This narrative review, synthesizing high-quality studies from diverse global regions, establishes HRV as a cornerstone for autonomic assessment in stroke patients, complemented by BRS, CARTs, pupillometry, BPV, and CAN screening (7, 8, 17, 18, 27, 28). Through conceptual synthesis, this discussion offers a deep analysis of clinical implications, integrates findings into a cohesive framework, provides practical recommendations grounded in clinical expertise, and outlines future directions from an expert perspective in stroke and autonomic research.

### Clinical implications in light of contemporary evidence (2022–2024)

Large-scale cohort studies reinforce the role of time-domain HRV as a reliable screening tool for early cardiovascular and functional risk (8, 9), while non-linear indices, such as sample entropy, demonstrate sensitivity in patients with high NIHSS scores or during structured rehabilitation (4, 29). A systematic review highlights HRV’s predictive value for stroke complications and outcomes, including cognitive impairment (16, 29). Frequency-domain features provide mechanistic insights into sympathovagal balance but require cautious interpretation due to methodological limitations, such as algebraic coupling of LFnu and HFnu, necessitating absolute values for accurate contextualization in acute instability (12, 14). Short-term BPV within the first 24 h post-stroke demonstrates superior prognostic accuracy compared to HRV alone, enhancing AUC in clinical models for recurrence and neurological deterioration (5–7). Beyond the hyperacute phase, combining nocturnal HRV with BPV and endothelial function improves prediction of recurrent cerebro-cardiovascular events, offering a template for long-term follow-up (26). Baroreflex sensitivity (BRS) serves as a prognostic marker for long-term outcomes and cardiovascular mortality, with studies highlighting its therapeutic potential (7, 27, 30, 31). Similarly, insular involvement in stroke increases arrhythmic risk, further emphasizing the role of autonomic assessment in complication prediction (2, 32). Recent trials also suggest that HRV biofeedback enhances rehabilitation outcomes by improving autonomic balance (33). Combining pupillometry with HRV improves prognostic accuracy for acute-phase outcomes, as demonstrated in recent multicenter studies (16). Frequency-domain features continue to provide mechanistic insights into sympathovagal balance, yet their interpretation now requires greater caution due to methodological pitfalls in normalized units, such as algebraic coupling of LFnu and HFnu, urging the use of absolute values for accurate contextualization in acute instability (14). BPV as a complementary early marker is gaining traction, with evidence suggesting that short-term and beat-to-beat BPV outperform HRV in predicting 90-day outcomes when measured within the first 24 h after ischemic onset, enhancing AUC in clinical models (16). Beyond the hyperacute phase, combining nocturnal HRV with BPV and endothelial function improves the prediction of recurrent cerebro-cardiovascular events, thus providing a practical template for long-term follow-up that incorporates vascular health assessments (7). This multi-signal approach highlights the need for tiered assessment models that adapt to the patient’s recovery stage, available resources, and clinical priorities, with recent systematic reviews confirming HRV’s broader role in complications and outcomes (33). Wearable technologies and AI-driven analytics are no longer experimental novelties: validated smartwatch-derived HRV metrics show near-parity with ECG gold standards for parameters like SDNN and RMSSD, making continuous autonomic monitoring feasible even outside specialized centers and supporting telerehabilitation pathways (11). This is particularly relevant for tele-rehabilitation and community-based follow-up, where early detection of autonomic deterioration can trigger timely clinical review, as demonstrated in feasibility studies with biofeedback-controlled exercise improving HRV at rest and during activity (11).

### Trends 2022–2024

A synthesis of the latest literature reveals several converging trends:

- *Hyperacute autonomic markers*: beat-to-beat BPV within 24 h is independently linked to poor 90-day mRS and enhances AUC when added to clinical models; beat-to-beat HRV alone is less consistent in that window (5, 16).
- *Early-phase HRV after reperfusion*: HRV dynamics in patients receiving thrombolysis relate to early neurological course and discharge outcomes, with parameter changes post-thrombolysis predicting deterioration (8, 9).
- *Multisignal risk stratification*: combining BPV + (nocturnal) HRV + endothelial function improves prediction of recurrent cerebro-cardiovascular events after ischemic stroke, offering refined vascular and autonomic insights (7).
- *Wearables/AI*: smartwatch-derived HRV shows high agreement with ECG for many metrics, enabling remote monitoring and telerehabilitation programs; feasibility data with biofeedback-controlled exercise in post-stroke cohorts are emerging (10, 11).
- *Methodological refinements*: 2024 critiques caution against over-interpreting LF/HF and normalized spectral indices without absolute values and careful contextualization, promoting standardization of influencing factors like posture and medication (12, 14).

### From data to practice: building tiered clinical pathways

Evidence from the past 6 years, sharpened by recent technological validation studies, supports a tiered ANS assessment strategy:

- *Acute phase (<24 h)*: short, high-quality HRV recordings (time-domain focus) supplemented by BPV measurement; if high risk is detected, consider BRS or pupillometry where available, with BPV providing superior short-term prognostic value (16).
- *Subacute and early rehabilitation*: incorporate non-linear HRV metrics and CARTs to detect persistent autonomic imbalance and tailor rehabilitation intensity, using biofeedback for controlled exercise to enhance HRV responses (11).
- *Long-term follow-up*: combine wearable-derived HRV, periodic BPV, and endothelial function assessment to monitor recovery trajectory and refine secondary prevention, with multi-signal models predicting recurrent events (7, 11).

### Balancing prognostic power and feasibility

When viewed through the dual lens of predictive accuracy and clinical accessibility, HRV and CARTs occupy the “high-value/high-feasibility” quadrant, making them foundational for broad implementation, especially with methodological standardization addressing LF/HF limitations (12, 14). BPV and pupillometry offer substantial prognostic gain but face equipment and training barriers, though pupillometry’s NPi < 3 threshold for delirium prediction can be integrated with HRV for improved triage (16). BRS remains largely confined to tertiary centers due to specialized requirements. Recent umbrella reviews on BPV emphasize its complementary role, while wearable validation expands HRV’s reach to telehealth, reducing barriers in resource-limited settings (5, 11). This balance between accuracy and practicality will underpin our proposed flowchart-based pathway and bubble chart comparison in the next section, aimed at guiding clinicians in tailoring ANS assessment to both patient needs and institutional resources. For example, in post-reperfusion scenarios, HRV dynamics can inform arrhythmic risk, while combining with pupillometry refines delirium prediction (8, 16).

### Which HRV metrics help when?

- *Time-domain (SDNN, RMSSD, pNN50)*: in the early post-acute days, reduced SDNN/RMSSD associates with worse neurological status and complications, including after thrombolysis; these measures are robust to brief artefacts and are easy to implement at bedside. They remain the practical first-line summary of global autonomic tone for ward-level risk screening, with recent data showing their predictive value post-thrombolysis (8, 9).
- *Frequency-domain (LF, HF, LF/HF)*: frequency features can capture sympathovagal shifts but are sensitive to respiration, total power, and normalization choices. 2024 methodological papers highlight algebraic coupling of normalized LF and HF (LFnu+HFnu = 1) and urge reporting absolute and normalized values together, avoiding simplistic “LF/HF = sympathetic/parasympathetic” interpretations—especially in acute physiological instability. Use frequency metrics to complement, not replace, time-domain indices, as critiques emphasize contextual factors like medication and posture (12, 14).
- *Non-linear (sample entropy, DFA)*: in moderate–severe strokes and during exercise/rehab, non-linear metrics capture complexity loss that may be missed by linear indices; early feasibility work shows HRV behavior under biofeedback-controlled workloads, informing individualized training intensity and improving outcomes in post-stroke cohorts (11).

### Practical integration with other biosignals

- *HRV + BPV*: in hyperacute ischemic stroke, beat-to-beat BPV is a stronger short-term outcome marker than beat-to-beat HRV; beyond the first day, nocturnal HRV + BPV (and endothelial function) meaningfully improve prediction of recurrent events over follow-up. *Implement*: capture 5-min beat-to-beat BP/HR traces in the first 24 h; then add 24-h or nocturnal HRV/BPV during step-down or early rehab, with umbrella reviews confirming BPV’s prognostic relevance (5, 7, 16).
- *HRV + Pupillometry (NPi)*: automated NPi on admission helps anticipate post-stroke delirium and neuroworsening; pairing NPi with HRV offers parallel windows into brainstem/ARAS function and cardiac-autonomic balance for deterioration triage, with NPi < 3 as a key threshold for high-risk identification (16).
- *HRV + Endothelial/vascular markers*: EndoPAT-derived endothelial function combined with HRV/BPV refines recurrent cerebro-cardiovascular risk after ischemic stroke—useful for tailoring antihypertensive and lifestyle prescriptions, as large cohorts show improved event prediction (7).

### Methodological notes for 2024+ protocols

1. Standardize recording context (posture, breathing, meds), report artefact handling, and include both absolute and normalized spectral values, as recent guidelines address influencing factors for reliable HRV measurement (12).
2. Use time-domain as baseline screening; add frequency/non-linear selectively (e.g., suspected sympathetic overactivity, exercise testing), with critiques warning against over-interpreting LF/HF without contextualization (14).
3. Where available, integrate wearable-derived HRV (validated against ECG) into telerehab dashboards; flag sudden SDNN/RMSSD drops for nurse call-backs, enabling remote monitoring in post-stroke recovery (11).

### Implementation challenges

Widespread adoption faces barriers, including methodological variability (e.g., HRV recording durations, CART protocols), equipment costs, and limited clinician awareness (29, 34). In resource-constrained settings, HRV and CARTs are feasible due to low costs, but advanced modalities like pupillometry ($5,000–$10,000) are restricted to urban centers (7, 35).

Lack of standardized guidelines, particularly for pupillometry and CAN screening, hinders integration, as Asian and US studies report variable thresholds (6, 30). Recent methodological papers emphasize the need for standardization to address these, including influencing factors like posture and medication in HRV protocols (12).

Clinician training is essential to ensure reliable CART and CAN assessments, with European studies noting inconsistent protocol application (36). Cost-effectiveness analyses are critical to justify investments in advanced tools, especially in regions like South America, where HRV wearables offer a scalable alternative with high ECG agreement (11, 35).

Global disparities in healthcare infrastructure require tailored implementation strategies, with rural areas prioritizing low-cost solutions and urban centers leveraging integrated platforms for biofeedback-enhanced rehabilitation (10, 20, 37). Expert perspective emphasizes stakeholder collaboration—clinicians, researchers, and policymakers—to address these barriers through training programs and standardized protocols, incorporating recent critiques on frequency-domain interpretation (14, 18).

### Future directions: an expert perspective

From the vantage point of autonomic and stroke research expertise, the following directions are proposed to advance ANS assessments:

1. *International standardization*: develop global guidelines for HRV and CART protocols to reduce variability and enhance reproducibility, building on European standardization efforts and recent methodological refinements (12, 34, 38, 49–53).
2. *Multi-center validation*: conduct large-scale, multi-center studies to validate pupillometry and CAN screening across diverse populations, addressing gaps in Asian and South American cohorts, including delirium prediction with NPi (7, 16, 18, 30).
3. *Longitudinal research*: investigate the longitudinal benefits of ANS-guided rehabilitation, particularly in understudied regions like South America and Eastern Europe, to establish long-term efficacy, with biofeedback protocols for exercise (10, 35, 39).
4. *Cost-effectiveness analyses*: evaluate the economic viability of HRV wearables and integrated platforms in resource-limited settings, supporting adoption in regions like Ukraine and Brazil, given their ECG validation (11, 35, 37).
5. *Global research networks*: foster collaborative networks to address geographic bias, integrating perspectives from underrepresented regions to enhance global applicability (20, 21).
6. *Emerging technologies*: leverage machine learning to integrate HRV, BPV, and pupillometry data for predictive modeling, potentially improving prognostic accuracy by 15–20%, as suggested by early studies (27). Recent cohorts in atrial fibrillation and post-thrombolysis settings provide data for such models (8, 13).
7. *Conceptual frameworks and tools*: develop pathophysiological diagrams and clinical decision algorithms to enhance clinician adoption. A pathophysiological diagram (see Pathophysiological_Diagram.md, Figure 1) illustrates the mechanistic links between stroke-induced ANS dysfunction and clinical outcomes, serving as an educational tool for clinicians and supporting the development of integrated assessment frameworks (18).

## Conclusion

This narrative review, synthesizing 53 high-quality studies from diverse global regions, establishes heart rate variability (HRV) as a cornerstone for autonomic assessment in stroke patients, complemented by baroreflex sensitivity (BRS), cardiovascular autonomic reflex tests (CARTs), pupillometry, blood pressure variability (BPV), and cardiac autonomic neuropathy (CAN) screening (1–8). HRV’s time-domain (SDNN, RMSSD, pNN50), frequency-domain (LF, HF, LF/HF), and non-linear metrics (sample entropy, DFA) provide unparalleled insights into cardiovascular risks, stroke recurrence, and functional recovery, making it a scalable tool for routine clinical use across acute and rehabilitation settings (9–11).

Recent methodological critiques emphasize the use of absolute versus normalized values for accurate frequency-domain interpretation, enhancing HRV’s reliability in unstable physiological conditions such as acute stroke (14). Integration with affordable wearable devices democratizes access, enabling risk stratification and personalized rehabilitation in both advanced stroke units and resource-limited clinics, such as those in Eastern Europe or South America (11, 12). Validation of smartwatch-derived HRV against ECG gold standards supports remote monitoring, with biofeedback protocols improving HRV during exercise and rehabilitation (10, 11).

Complementary modalities enhance prognostic precision: BRS and BPV guide cardiovascular management, with BPV excelling in hyperacute 24-h predictions (5, 7, 16); CARTs and pupillometry offer rapid bedside insights, where NPi < 3 predicts delirium and integration with HRV refines triage (16–20); and CAN screening targets high-risk patients with comorbidities (6). This work advances the field by consolidating global evidence, addressing methodological challenges through standardization guidelines (12, 30), and proposing practical strategies for clinical adoption.

Despite limitations like protocol variability and geographic bias, the robust evidence base supports integrating multi-modal ANS assessments into stroke care. We advocate for international guidelines to standardize protocols, multi-center validation studies for emerging modalities like pupillometry in delirium prediction (16), and cost-effectiveness analyses to bridge implementation gaps, paving the way for transformative improvements in stroke outcomes worldwide (11, 35, 37).
